# Evaluating treatments in health care: The instability of a one-legged stool

**DOI:** 10.1186/1471-2288-11-65

**Published:** 2011-05-11

**Authors:** Bonnie J Kaplan, Gerald Giesbrecht, Scott Shannon, Kevin McLeod

**Affiliations:** 1 Department of Paediatrics, Faculty of Medicine, University of Calgary, Calgary, Alberta, Canada; 2 Department of Psychiatry, University of Colorado, Denver, Colorado, USA; 3 Bachelor of Health Science Program, Faculty of Medicine, University of Calgary, Calgary, Alberta, Canada

## Abstract

**Background:**

Both scientists and the public routinely refer to randomized controlled trials (RCTs) as being the 'gold standard' of scientific evidence. Although there is no question that placebo-controlled RCTs play a significant role in the evaluation of new pharmaceutical treatments, especially when it is important to rule out placebo effects, they have many inherent limitations which constrain their ability to inform medical decision making. The purpose of this paper is to raise questions about *over-reliance *on RCTs and to point out an additional perspective for evaluating healthcare evidence, as embodied in the Hill criteria. The arguments presented here are generally relevant to all areas of health care, though mental health applications provide the primary context for this essay.

**Discussion:**

This article first traces the history of RCTs, and then evaluates five of their major limitations: they often lack external validity, they have the potential for increasing health risk in the general population, they are no less likely to overestimate treatment effects than many other methods, they make a relatively weak contribution to clinical practice, and they are excessively expensive (leading to several additional vulnerabilities in the quality of evidence produced). Next, the nine Hill criteria are presented and discussed as a richer approach to the evaluation of health care treatments. Reliance on these multi-faceted criteria requires more analytical thinking than simply examining RCT data, but will also enhance confidence in the evaluation of novel treatments.

**Summary:**

Excessive reliance on RCTs tends to stifle funding of other types of research, and publication of other forms of evidence. We call upon our research and clinical colleagues to consider additional methods of evaluating data, such as the Hill criteria. Over-reliance on RCTs is similar to resting all of health care evidence on a one-legged stool.

## Background

The fact that so many pathological syndromes are named after the individual who first characterized the disorder illustrates that medicine has always valued good clinical observations. In fact, one could argue that most major discoveries in health have evolved from observations first documented in case reports and case series. Randomized controlled trials (RCTs) are often mounted later to validate an intervention, especially in comparison with a placebo, but as is well-recognised, they are rarely major sources of scientific discovery. In spite of the fact that observational data hold a time-honored place in medicine, 21^st ^century methodology has pre-empted several millennia of historical tradition by anointing RCTs with the descriptive phrase 'gold standard' of evidence [1].

There are many reasons why RCTs have become the *de facto *standard by which all forms of evidence are evaluated. No other study design rivals the RCT's ability to eliminate selection bias and reduce the risk of a serious imbalance in known and unknown factors that could influence outcomes (when the randomization procedure is executed properly). Our concern is that evidence based medicine has made a leap from considering RCTs to be a high standard to being the *only *standard. The primary purpose of this paper is to question the over-valuation of RCTs that Rosenbaum referred to as a form of tyranny [2]. Our premise is that over-reliance on RCTs has resulted in a foundation for decision-making in health care that is as unstable as a one-legged stool. The history and limitations of RCTs are first summarized and then followed by suggestions for an alternative approach to the evaluation of evidence. The second purpose of this paper is to propose greater use of alternative forms of evidence. Depending on a variety of sources leads to more stable and error-free conclusions by providing other legs to stabilize the stool upon which medical decision-making can rest.

### History of RCTs

The use of randomized controlled trials predates the actual term RCT by several centuries. One of the earliest documented applications to health was the proof that citrus prevented scurvy [3]. Scurvy routinely killed > 50% of sailors on long voyages, no small impediment to world exploration in the 14^th^-18^th ^centuries. A dietary factor was suspected in 1601 when Captain James Lancaster administered a tablespoon of lemon juice per day to each sailor on one ship: 0% of those on the ship with lemon juice rations died, while 40% of those on the three ships without lemon juice were dead halfway through the journey to India. Replications and extensions followed: e.g., in the mid-18^th ^century a ship physician named James Lind conducted a study in which early signs of scurvy were effectively treated in those randomly assigned to receive citrus, thereby showing the ability of citrus to reverse the disease in its early stages [3].

It was Sir Austin Bradford Hill (sometimes referred to as Bradford Hill, or Bradford-Hill), a British statistician and epidemiologist, who promoted the use of randomization for clinical trial research employed to test health care interventions, a position he took prior to World War II [4]. However, the issue became more prominent in 1946 when the British Medical Research Council was investigating the effect of streptomycin on tuberculosis. The extreme shortages of streptomycin in England caused considerable stress amongst physicians who were constrained to use existing therapies despite reports of a promising new intervention. The consensus at the time was that the "small supply of streptomycin allocated to it for research purposes would be best employed in a rigorously planned investigation with concurrent controls" (page 769) [5].

In 1962, RCTs were still quite rare, yet they were the norm in 1992 when the Evidence-Based Medicine Working Group published their seminal paper [6]. The escalation of the importance of RCTs in this 30-year period was influenced worldwide by significant decisions made in the United States regarding premarket approval of drugs by the Food and Drug Administration (FDA). It was section 355(d) of the 1962 Drug Amendments to the American Food, Drug, and Cosmetic Act which changed procedural requirements in the United States [7]. This clause was the first time FDA introduced the requirement of what they referred to as 'effectiveness' for its approval, in addition to the previous requirement of safety, a change that led directly to incorporating randomization and blinding into studies [7]. According to Kulynych's historical review, consideration of public safety was the basis of the requirement for effectiveness: if an ineffective drug replaces one of proven value, people can be harmed. Thus, even though the primary mandate had previously been safety of drugs, the rationale that led in 1962 to section 355(d) evolved from recognition of the importance of effectiveness for the demonstration of safety. Litigation that followed the Drug Amendment clarified that RCTs would be required as proof of efficacy, and hence meeting the safety requirement. The new statute also required that pharmaceutical companies provide "substantial evidence" consisting of "adequate and well-controlled investigations, by experts qualified by scientific training" to demonstrate effectiveness of a new drug. Subsequent legal interpretation clarified that two RCTs would be required to demonstrate that effectiveness [7].

The new effectiveness criterion had a significant impact on standards of scientific evidence for the next 30 years; however, by the end of the 20th century, even FDA was beginning to question the need for multiple Phase III RCTs as proof of effectiveness to justify market approval [7]. Criticisms were based primarily on cost and inefficiency: clinical trials for market approval cost millions of dollars. In the 1997 Modernization Act of FDA, the requirement of two RCTs was softened to one, but RCTs continued to be the gold standard for market approval of drugs.

Since the era of Hill's methodological contributions, various groups have promoted the idea of levels of evidence, but recently, some have questioned the position of RCTs in a hierarchical model. For instance, Ghaemi commented that "...the key feature of levels of evidence to keep in mind is that each level has its own strengths and weaknesses, and as a result, no single level is completely useful or useless" (p.10) [8]. Further, Walach and colleagues emphasized that the RCT hierarchy of evidence is based on the pharmacological model of treatment, and is not always appropriate for the evaluation of interventions [9]. They argued for a Circular Model, based on many methodologies and designs -- what might be considered a return to the historical principle of depending on the 'weight of the evidence.' The Circular Model poses the idea that experimental methods (such as RCTs) used to evaluate efficacy need to be complemented by other methods that take into account real-life issues and clinical applicability. As Walach and colleagues conclude, "Rather than postulating a single "best method'' this view (the Circular Model) acknowledges that there are optimal methods for answering specific questions, and that a composite of all methods constitutes best scientific evidence." Some areas of science refer to this as a 'multi-method' approach.

### Limitations of RCTs

RCTs bolster confidence in causal claims related to the effects of a treatment by eliminating threats to internal validity. They do this by using the tools of random assignment and experimental control. However, a medical science that relies primarily upon achieving internal validity with a relative neglect of external validity (as, we argue, many RCTs do) is at great danger of ignoring the individual and context characteristics that impinge upon treatment outcome. The five criticisms of RCTs reviewed below redirect us to consider a more diverse medical science.

1. *RCTs usually lack external validity*. For any given study, clinicians should reasonably ask, to whom do these results apply? Particularly in mental health, it has been shown that RCTs tend to employ such strict inclusion and exclusion criteria that the participants are not representative of the general population of individuals with a given disorder. As Concato and colleagues pointed out [10] (p. 1891): "...an observational study would usually include patients with coexisting illnesses and a wide spectrum of disease severity." But the most typical characteristic that excludes people from RCTs is just that - the presence of a co-existing disorder [11,12]. Deisboeck [13] observed (p. 2) that "the status quo strategy in medical practice is as simple as it now appears to be intrinsically flawed: carefully assess a patient's symptoms to diagnose his specific disease patterns only to then treat it with a protocol that is based on the assumption that most interpersonal characteristics are rather inconsequential for treatment outcome."

The external validity (or ecological validity) and generalizability of RCTs have been questioned for a number of years [14], and a recent report provided an elegant test of the issue in the context of psychiatry. The study known as the Sequenced Treatment Alternatives to Relieve Depression (STAR*D) is a well-known, multi-centre prospective trial of medications for major depressive disorder [12]. Adults with major depressive disorder, and no history of bipolar, psychosis, eating disorder, or obsessive compulsive disorder, were invited to participate in a study that began with an evaluation of citalopram. The investigators analyzed data collected on almost 3,000 STAR*D participants to address the question of whether Phase III RCTs are studying patients who are representative of depressed outpatients in the population. Using standard clinical trial criteria, they separated participants into those who met the inclusion criteria for a Phase III RCT (N = 635) and those who did not (N = 2,220). In other words, only 22% of the STAR*D participants would have passed screening for a traditional RCT. So in answer to the question raised above ('to whom do these results apply?'), it appears that the information obtainable from a traditional RCT with this sample would not have been directly relevant for 78% of people suffering from major depressive disorder. The STAR*D trial, with its generally more inclusive eligibility criteria, is actually an example of how external validity can be improved in RCTs.

*2. In the long term, RCTs may actually increase health risks in the general population*. As a corollary to the issue of external validity, excessively constrained sampling approaches can have consequences for population health. Health researchers sometimes distinguish between efficacy and effectiveness. One might say that all RCTs are efficacy studies (though the terminology becomes confusing, as they are submitted to FDA as evidence of effectiveness as spelled out in section 355(d)), demonstrating benefit in ideal conditions - particularly by selecting only relatively 'pure' samples of people with no co-existing problems. The lack of external validity caused by these atypical samples means that the drugs are approved without evidence of effectiveness (defined as benefit in the broader population, under less ideal circumstances). Since people with complex health problems (e.g., hypertension or heart disease) are not usually participants in RCTs evaluating mental health treatments, it is reasonable to consider the possibility that patients prone to adverse effects are not studied. But approval of the drug then has the potential of increasing the risk of those vulnerable individuals to exacerbation of their pre-existing health problems. One could argue that effectiveness studies using designs such as case-control methodology that have more external validity would be more informative about the health impacts of a new drug for the broader population. Without such forms of 'observational' studies, drug approval based only on RCTs may be increasing the health risks to the populace.

3. *The premise that RCTs are the only form of evidence capable of providing an unbiased estimate of treatment effects is false*. A fundamental reason for the elevated status of RCTs is the conviction held by many that all other forms of evidence, even cohort and case-control studies, overestimate treatment effects. However, some published research does not support this premise. Concato and colleagues [10] evaluated 99 reports of five distinct clinical topics and could find no meaningful differences in the treatment effects on a broad array of clinical outcomes obtained from RCTs compared to observational data. As they pointed out, the literature on psychological, educational, and behavioral treatments have revealed similar findings: no difference in effect magnitude reported from RCTs vs. observational studies [15]. So even though randomization, blinding, and placebo controls contribute to a high degree of internal validity, requiring all evidence to fit this model appears to be unjustified.

4. *RCTs are unable to tell clinicians everything they really want to know*. Comparisons between groups of individuals can obscure processes operating within individuals. However, RCTs only reveal differences between treatment and control group means - these aggregated results are uninformative of the potential benefit of a treatment for any individual in the study, and more importantly, for the individual who was not in the study but whose treatment decisions will nevertheless be made on the basis of the study [1]. What clinicians really want to know is whether or not the person sitting before them is likely to benefit. The averaged results derived from RCTs offer insufficient or even incorrect guidance on how to approach a specific case [13]. There is no doubt that RCTs provide high quality information about treatments which should be considered, especially where stratified groups have been included in the analysis. Nevertheless, additional forms of evidence that explicitly include individual and context characteristics are needed to assist clinicians in choosing a course of action regarding specific patients. Single case experiments, epidemiological data, qualitative data and field reports from clinicians using an intervention are examples of such additional sources of information.

*5. The excessive expense of RCTs leads to vulnerabilities in the quality of evidence*. On average, a Phase III clinical drug trial costs > 15 million USD [16], which raises several important issues.

• Part of the cost of an RCT in some countries is significant payment to participants, which has led to a practice of 'guinea-pigging' [17-19] in which some people volunteer for research to gain money or free physical exams. In mental health research it is easy for people to fake symptoms to gain access to these rewards, as there are no biomarkers of mental disorders. One of many problems with this practice is that there is no oversight body to ensure that people have not recently participated in some previous RCT; hence, there is no assured washout period between exposures to different drugs.

• The expense of RCTs adds to the cost of medications that are eventually approved. Hence, the broader population has the right to ask if they should be paying for research that has questionable generalizabilty to the general populace.

• The significant costs of RCTs add pressure to bury negative reports that will prevent a medication from moving to market, thereby removing the possibility of development cost recovery by the pharmaceutical company. The total cost of developing a new drug is now in excess of US$1.3 billion [16] - a substantial investment to lose in the case where the promise of a new drug is not realized. The analysis by Turner and colleagues highlighted this concern [20] by analyzing publication bias in the literature on anti-depressants. In 74 clinical trials of antidepressants, 37 of 38 positive studies were published. But of the 36 negative studies, 33 were either not published or published in a form that conveyed a positive outcome. Such evidence for a strong and consistent commercial distortion in the medical data base used to support evidence-based medicine is very worrisome. Without access to published reports that fail to demonstrate treatment efficacy, the weight of evidence becomes biased in favor of the treatment. Clinical trial registration systems are, of course, beginning to address this problem.

• The expense of RCTs biases their implementation toward areas for which commercial funding is possible; i.e., pharmaceutical interventions. Million dollar grants are less available for the evaluation of non-conventional forms of treatment (natural health products, acupuncture, psychotherapy, etc.). When the valuation of RCTs as "gold standard" is combined with a systematic bias toward commercial applications, the weight of evidence itself becomes biased in favor of pharmaceutical treatments.

• A final adverse side effect of the high costs of RCTs, at least with regard to psychiatric drugs, is the brief intervention period that is typically employed to evaluate efficacy. Most RCTs in mental health now last only 6-8 weeks, which is more economical than a 12-week or longer trial more typical a decade ago (see, for example [21]). But clinicians have to make decisions about long-term treatment for their patients, usually based on this very short-term information. An additional weakness that this trend imposes is that safety issues resulting from long term use are often unknown.

The unfortunate result of these financial pressures is that clinical decisions are made to administer medications to patients for many years, but the decisions themselves may be based on very brief trials conducted with very unusual people (with no other health problems, for instance), and without evidence for other interventions which may be effective.

In conclusion, dismissing or devaluing rigorously collected data obtained with study designs other than RCTs results in a science that is inherently unsupportable - as shaky as a one legged stool. Indeed, as Concato and colleagues argued, the evidence does not support the commonly accepted concept of a hierarchy of study designs employed for clinical research [10]. If RCTs are not the only or even the most important evidence for evaluating effectiveness, then we need to ask what other criteria we can use to support a rigorous evidence-based medicine. This question brings us full circle, back to Sir Austin Bradford Hill.

### The Nine Hill Criteria for Defining Causality

Hill was primarily concerned with causation of disease when he outlined nine considerations for determining causal relations in epidemiology, using the association between smoking and lung cancer as his illustration. However, the criteria he defined in his 1965 presidential address to the Section of Occupational Medicine of the Royal Society of Medicine [22] are also employed to evaluate causal explanations in other contexts [23]. The criteria will be addressed with special reference to mental health issues in order to illustrate their potential utility for evaluating treatments in an area of clinical science where medical and non-medical approaches are often combined.

1. *Strength*. The strength of association between an outcome and a putative causative agent is an important signal of a causal relationship. All things being equal, a strong association is less likely to occur from extraneous than causal effects. Hill did caution that no matter how slight an association may be, it should not be dismissed until argument for or against causality exists, and he used as an illustration the evidence that relatively few persons harboring meningococcus actually become ill with meningococcal meningitis. As discussed above (item 4), the fact that a causal relation between two variables can be detected within persons but are not observed when data are aggregated should caution against methods that rely exclusively upon indices of group differences. Therefore, methodology that relies solely on mean differences is a limited approach, and alternatives that accommodate individual differences may bolster conclusions. Such alternatives might include qualitative methods [1] or within-subject crossover designs that are able to demonstrate on-off control of symptoms in subgroups of the sample.

Defining the strength of association between treatments and symptom severity has been an especially contentious issue in psychiatry, particularly with respect to depression. There has been much concern that publication bias against negative findings, discussed above, results in approval of medications in spite of multiple trials failing to show benefit over placebo. Using Freedom of Information legislation to gain access to unpublished studies of anti-depressant efficacy, Kirsch and colleagues showed very little difference between medication and placebo in 35 RCTs on four SSRIs [24]. They used symptom change on the Hamilton Rating Scale of Depression (HRSD) as the outcome measure, and confirmed previous findings: there was an improvement of 9.6 points for the medication group and 7.80 for placebo controls. Although this 1.8 point spread is statistically significant, it is below 3.0, the value necessary for clinical significance used by the National Institute for Clinical Excellence (NICE).

*2. Consistency*. Hill used this term to refer to obtaining similar results across different research sites and methodologies, something we might now call replication. As he pointed out, repeating studies is necessary to prove the obtained association is not a result of confounding variables in one setting or group. Similar results from independent researchers using different methods are more convincing than a single study. In the social sciences, this is generally referred to as method triangulation, or multi-methods [9].

*3. Specificity*. Hill recognized that two different patients may have varied outcomes from treatment simply based on individual variables. Accordingly, it is not always possible to demonstrate specificity, even when a causal association exists. For example, Hill noted that smokers have a higher death rate than non-smokers for many causes of death. However, the relative increase for other diseases is modest (10-20%) while the increase for lung cancer is 900 - 1000%. Such specificity in the *magnitude *of the association provides important evidence for a causal association. In contrast, there are multiple determinants of mental disorders (psychosocial, biological, societal), making it very difficult to estimate the specific contribution of any particular predictor variable. Because of this multicausality, this criterion may not always be applicable to evaluating causality in areas such as mental health, where family dynamics and other social issues can play such a prominent role. Qualitative methods may also play a role in the generation of hypotheses in such situations.

*4. Temporality*. Temporality refers to the common sense notion that the cause always precedes the outcome. Temporality is crucial for determining direction of causality: e.g., does the decrease in a factor result in a disease, or does the disease result in the decrease of that factor? In mental health, elucidating this relationship can be difficult, but not impossible. For instance, within-subject crossover study designs (e.g., ABAB where A is the active treatment and B is a period of placebo, or at least removal of the active treatment) can be useful for investigating the effect of a treatment. Assuming the existence of minimal carryover effects and sufficient time devoted for washout, this methodology can show (a) whether there is an improvement in the patient's condition, and (b) whether the treatment was actually the factor causing the improved outcome and not a confounding variable. We note that when it is possible to randomly allocate the treatment sequence, such cross-over designs can be considered RCTs.

*5. Biological Gradient*. A biological gradient is best described as a dose-response curve: increased treatment would presumably result in a proportionate increase in the effect. Hill realized that this criterion might not be applicable to all research fields, and he recommended that it be considered only when logical. Within psychiatry, outcomes vary largely because of individual differences. For example, it is clear that the optimum dose of a given medication is not necessarily the highest one tested. Even in the Kirsch meta-analysis described above, the relationship between severity and response to medication was not linear [24]. Where the biological gradient for a disorder is complex (as in mental health), this criterion is not necessarily applicable for establishing causality.

*6. Plausibility*. Plausibility refers to whether the cause and effect can be reasonably connected given the current state of knowledge within the discipline. Importantly, Hill states that new findings must not be immediately dismissed if they do not fit in with current knowledge ("dogma") on the subject. As Kuhn observed, considerable evidence disconfirming the accepted view must accumulate before new ways of thinking can emerge from new data [25]. Furthermore, a new treatment could alleviate symptoms of a disorder, but be disregarded because its mechanism of action may be unknown.

*7. Coherence*. Similar to plausibility, coherence refers to the agreement of a study's findings with what is already known. According to Hill, the cause-effect interpretation of the data should not seriously interfere with current knowledge of the disease. Furthermore, Hill mentioned that basic lab evidence should not be a requirement, primarily because some outcomes would be difficult to demonstrate in a controlled environment. For instance, the search for animal models of human mental disorders has a long history, but no matter how promising some of the achievements, science can never validate an animal model for internal mental functions such as delusions, suicidality, euphoria, or hallucinations. For this reason, the criterion of coherence may not be a requisite standard for evaluating a novel approach to something like mental disorders.

*8. Experiment*. The criterion of experimental evidence can be fulfilled in many ways. One reason for the wide acceptance of RCTs for pharmaceuticals is the well-documented placebo/expectancy effect in psychiatry. As Kirsch et al. commented regarding their meta-analysis, the longer-term improvements for medication observed in those 35 RCTs seemed to be a result of the decreasing placebo response which had been quite high (> 80%) [24]. Placebo-controlled randomized trials provide one type of design to control for placebo effects. Other alternatives include within-subject crossover designs and case-control studies.

*9. Analogy*. The notion that a similar cause results in a similar outcome is referred to as analogy. Hill described this criterion as accepting less evidence based on previous results, citing the case of pregnancy and thalidomide. If a new drug were to demonstrate negative consequences during pregnancy, we would be less hesitant to stop its use even if little evidence of harm exists, because of the tremendous social and personal cost that resulted from thalidomide. This criterion may not be relevant to all areas of health care treatment, particularly mental health. As with the criteria of specificity and biological gradient, the issue comes down to the multicausality of mental disorders and the multifinality of patient outcomes. Two patients presenting with similar illness sometimes respond quite differently to an identical treatment. Likewise, the justification that a past patient responded positively to a drug does not ensure that the current patient will, which introduces uncertainty about the nature of the association between the treatment and the outcome.

Hill emphasized that not all nine criteria would be applicable to all situations. For instance, five appear to be applicable for the demonstration of causality in mental health: Strength, Consistency, Temporality, Plausibility, and Experiment. For any application, common sense needs to prevail when considering criteria to evaluate causality.

## Discussion

One could argue that over-reliance on RCTs has fostered a less critical form of thinking in the evaluation of health care treatments. Several years ago Smith and Pell wrote a satirical, insightful commentary on the need to do an RCT of the effectiveness of parachutes for the prevention of major trauma caused by gravity [26]. They concluded that people "...who insist that all interventions need to be validated by a randomised controlled trial need to come down to earth with a bump" (p. 1460). We suggest that ignoring data from sources other than RCTs results in a one-legged stool that brings progress in health treatment down with a bump.

The methods we use constrain the types of observations we can make. Because of this, it is important to use as many different sources of information as possible. Multi-method research can provide converging evidence on treatment effects, where "multi-method" refers to obtaining diverse sources of information that are minimally related to the existing sources. Unfortunately, it is increasingly difficult to fund or publish studies that are not RCTs. While the majority of social scientists fly the multi-method banner, it is RCTs that primarily hold the attention of health researchers. This dependence on RCTs means that the weight of evidence is precariously balanced upon a single method, a clear example of the instability of a one-legged stool. Because inferences from clinical research propagate to clinical practice, failure to consider multiple sources of information compromises the foundation on which medical decisions are based, and on which the fate of lives may rest.

## Summary

In summary, over-reliance on RCTs a) has been influenced in part by market pressures relevant to pharmaceutical companies, b) was stimulated significantly by the 1962 amendments to the American Food, Drug, and Cosmetic Act, and c) is not scientifically sound. As Parker stated (p. 971) [1], "...it seems imprudent to assume that one type of methodology provides the only path to knowledge." There are alternatives to depending solely on RCTs, especially from the perspective provided by the Hill criteria, which enable us to more fully evaluate treatments in health care.

## Competing interests

The authors declare that they have no competing interests.

## Authors' contributions

BJK, SS, and KM conceived the manuscript and drafted it. GG and BJK provided the major portion of shaping the final content. All authors edited multiple versions and approved the final manuscript.

## Authors' information

BJK and GG are research psychologists; SS is a clinical and academic psychiatrist; KM is a graduate student in the Health Sciences Program, with a special interest in clinical research methodology.

## Pre-publication history

The pre-publication history for this paper can be accessed here:

http://www.biomedcentral.com/1471-2288/11/65/prepub
